# miR-146a-Enriched Mesenchymal Stem Cell-Derived Extracellular Vesicles Prolong Corneal Allograft Survival by Modulating Local and Systemic Immune Responses

**DOI:** 10.5812/ijpr-171093

**Published:** 2026-05-30

**Authors:** Xuting He

**Affiliations:** 1 Department of Ophthalmology, Xi 'an Hospital of Traditional Chinese Medicine, Xi 'an, China

**Keywords:** Cytokines, Immune Response, MicroRNA, Transplantation

## Abstract

**Background:**

Corneal allograft rejection remains a leading cause of graft failure. Mesenchymal stem cell-derived extracellular vesicles (MSC-EVs) may provide new options for modulating the immune response to allografts.

**Objectives:**

This study aimed to determine whether miR-146a-enriched EVs could prolong corneal allograft survival by modulating local and systemic immune responses.

**Methods:**

After corneal allografting from C57BL/6 to BALB/c mice, recipients received subconjunctival injections of PBS, miR-NC-EVs, or miR-146a-EVs isolated from human umbilical cord mesenchymal stem cells (hUCSCs). Local inflammatory and regulatory factors in ocular tissues were assessed using quantitative real-time polymerase chain reaction (qRT-PCR) to quantify the expression of interleukin-1 receptor-associated kinase 1 (IRAK1), TNF receptor-associated factor 6 (TRAF6), tumor necrosis factor (TNF)-α, and forkhead box protein 3 (Foxp3). Systemic cytokine profiles were assessed in recipient sera using enzyme-linked immunosorbent assay (ELISA) to measure interferon (IFN)-γ, interleukin (IL)-17, IL-10, and transforming growth factor (TGF)-β.

**Results:**

Clinical rejection scores were markedly reduced, with significant improvements in opacity, edema, and neovascularization. RT-PCR analysis of corneal tissue showed miR-146a overexpression, downregulation of IRAK1 and TRAF6, a significant reduction in TNF-α mRNA, and increased Foxp3 expression. ELISA analysis of systemic IFN-γ and IL-17 showed that treatment with miR-146a-EVs resulted in lower systemic IFN-γ and IL-17 levels and significantly higher levels of the anti-inflammatory cytokines, IL-10 and TGF-β.

**Conclusions:**

This study identifies miR-146a-EVs as a novel cell-free therapy that effectively prolongs corneal transplant survival. The treatment exerts dual immunomodulatory effects by regulating local inflammatory pathways and shifting systemic cytokine profiles toward an anti-inflammatory state, thereby offering a targeted strategy to prevent transplant rejection.

## 1. Background

Corneal transplantation is one of the most commonly performed tissue transplantation procedures worldwide (1). Keratoplasty is performed to restore vision in many ocular diseases. Although corneal transplantation is routinely performed, it is often overlooked in broader transplantation science, despite substantially improving patients' quality of life through vision restoration (2).

The initial step in corneal allograft rejection is antigen processing by host antigen-presenting cells (APCs) and presentation of these antigens to host T cells, producing an allospecific immune response that mediates rejection. This response is enhanced in inflamed corneas by neovascularized blood vessels and aberrant cytokine production. Therefore, local or regional immunosuppression may help abrogate the heightened response associated with corneal transplant rejection. Access to the cornea also enables the topical application of therapies, such as monoclonal antibodies, gene therapy, or mesenchymal stem cell-derived extracellular vesicles (MSC-EVs), that may interfere with the local response to the transplanted cornea and reduce the adverse effects of systemic immunosuppression.

Mesenchymal stem cells (MSCs) are self-renewing, pluripotent, nonhematopoietic stem cells that can differentiate into multiple mesodermal lineages and modulate immune responses. Therefore, they have been identified as a viable strategy for regenerative medicine (3). More recently, MSC-EVs have been recognized as potential agents for modulating immune function in several inflammatory and transplantation settings, particularly in corneal regeneration (4, 5). Extracellular vesicles carry important cargo, including proteins, lipids, and microRNAs (miRNAs), that mimic many immunoregulatory actions of MSCs (6).

miR-146a is one of the most extensively studied miRNAs derived from MSC-EVs because of its potential to mediate and regulate innate immune responses (7, 8). miR-146a regulates innate immunity through its effects on major targets in the toll-like receptor (TLR) pathway and cytokine signaling pathways, including interleukin-1 receptor-associated kinase 1 (IRAK1) and TNF receptor-associated factor 6 (TRAF6) (9). Therefore, miR-146a serves as a negative feedback regulator that downregulates TLR- and cytokine-associated inflammatory responses through inhibition of nuclear factor kappa B (NF-κB) and downregulation of pro-inflammatory cytokine expression (10).

Considering these characteristics, vesicular delivery of miR-146a may provide a therapeutic opportunity for corneal transplant rejection driven by excessive local inflammation and abnormally high pro-inflammatory cytokine levels that interfere with optimal healing and recovery. However, the efficacy of MSC-EVs enriched with miR-146a in preventing corneal allograft rejection remains to be elucidated.

## 2. Objectives

This study aimed to evaluate the therapeutic potential of miR-146a-enriched human umbilical cord MSC-EVs (hUCSC-EVs) administration to promote corneal graft survival, modulate local inflammatory responses, and regulate systemic cytokine levels.

## 3. Methods

### 3.1. Ethical Considerations

All animal experimental procedures were conducted in full compliance with the ARRIVE (Animal Research: Reporting of In Vivo Experiments) guidelines and in accordance with the ethical standards outlined in the Guide for the Care and Use of Laboratory Animals (National Academy of Sciences, National Institutes of Health Publication No. 86 - 23, revised 1985). Human research was conducted in accordance with the principles of the Declaration of Helsinki and local regulatory requirements. All umbilical cord samples were collected from adult donors who provided written informed consent. All animal and human experiments were approved by the Ethics Committee of Xi'an Hospital of Traditional Chinese Medicine (Ethical No.: ET20250519), and all procedures were performed in accordance with the institution's guidelines.

### 3.2. MSC Isolation From the Human Umbilical Cord and Characterization

Approximately 20 cm of umbilical cord was collected from healthy mothers who provided informed consent after first-term delivery. Samples were transported to the laboratory within 6 hours of collection and processed to isolate human umbilical cord MSCs (hUCSCs). Umbilical cords were washed with phosphate-buffered saline (PBS), cut into 2-cm segments, and compressed to remove residual blood. The cord segments were minced into 1- to 3-mm cubes and digested for 1 hour using type II collagenase and 0.125% trypsin in PBS. The digestion solution was neutralized with fetal bovine serum (FBS; 10%), and the cell pellet was collected by filtration and centrifugation. The pellet was resuspended in low-glucose Dulbecco's Modified Eagle Medium (L-DMEM) containing 10% FBS, penicillin (100 U/mL), and streptomycin (100 μg/mL). After seeding into T75 culture flasks, cells were incubated at 37°C with 5% CO_2_. The medium was changed every 3 days until cultures reached approximately 80% confluence, at which time passaging was performed (11).

Isolated cells were identified as MSCs according to accepted criteria. Using flow cytometry, cells were assessed for the surface markers CD90 and CD105 and the hematopoietic lineage marker CD45.

The in vitro multilineage differentiation potential of the isolated cells was evaluated. Adipogenic differentiation was induced for 2 weeks in induction medium containing 1 × 10^-8^ M dexamethasone and 10 ng/mL insulin and was assessed using Oil Red O staining. Osteogenic differentiation was induced for 3 weeks using osteogenic medium containing 10 mM glycerol 2-phosphate disodium salt, 1 × 10^-8^ M dexamethasone, and 50 μg/mL L-ascorbic acid-2 phosphate and was assessed using Alizarin Red S staining.

### 3.3. Transfection of hUCSCs with miR-146a

Transfection of hUCSCs was performed when cells reached approximately 80% confluence. Cells were transfected using Lipofectamine 3000 and Opti-MEM and incubated for 72 hours according to the manufacturer's instructions at a 1:100 ratio with miR-146a mimics and negative control (NC) mimics.

### 3.4. EV Isolation and Characterization

Differential ultracentrifugation was used to isolate EVs from hUCSCs transfected with either miR-146a mimics or control mimics in EV-depleted conditioned medium. Conditioned medium was harvested after 48 hours of culture in exosome-depleted serum and subjected to sequential centrifugation at 300 × g for 10 minutes, 2000 × g for 10 minutes, and 10000 × g for 30 minutes, followed by final double pelleting at 100000 × g for 70 minutes. The isolated vesicle pellet was resuspended in sterile PBS.

To characterize the isolated EVs, levels of the phenotypic marker CD9 were analyzed by flow cytometry, and protein concentrations were determined using the bicinchoninic acid (BCA) protein assay kit (Solarbio, China). Dynamic light scattering (DLS) analysis using a HORIBA SZ-100 instrument was performed to assess the EV size distribution and mean diameter. Samples were prepared by resuspension in PBS, maintained at 25°C, and analyzed at a 173° scattering angle with a 30% neutral density filter. The instrument software (version 2.20) was used for data analysis.

### 3.5. Animal Model

**Figure 1.**
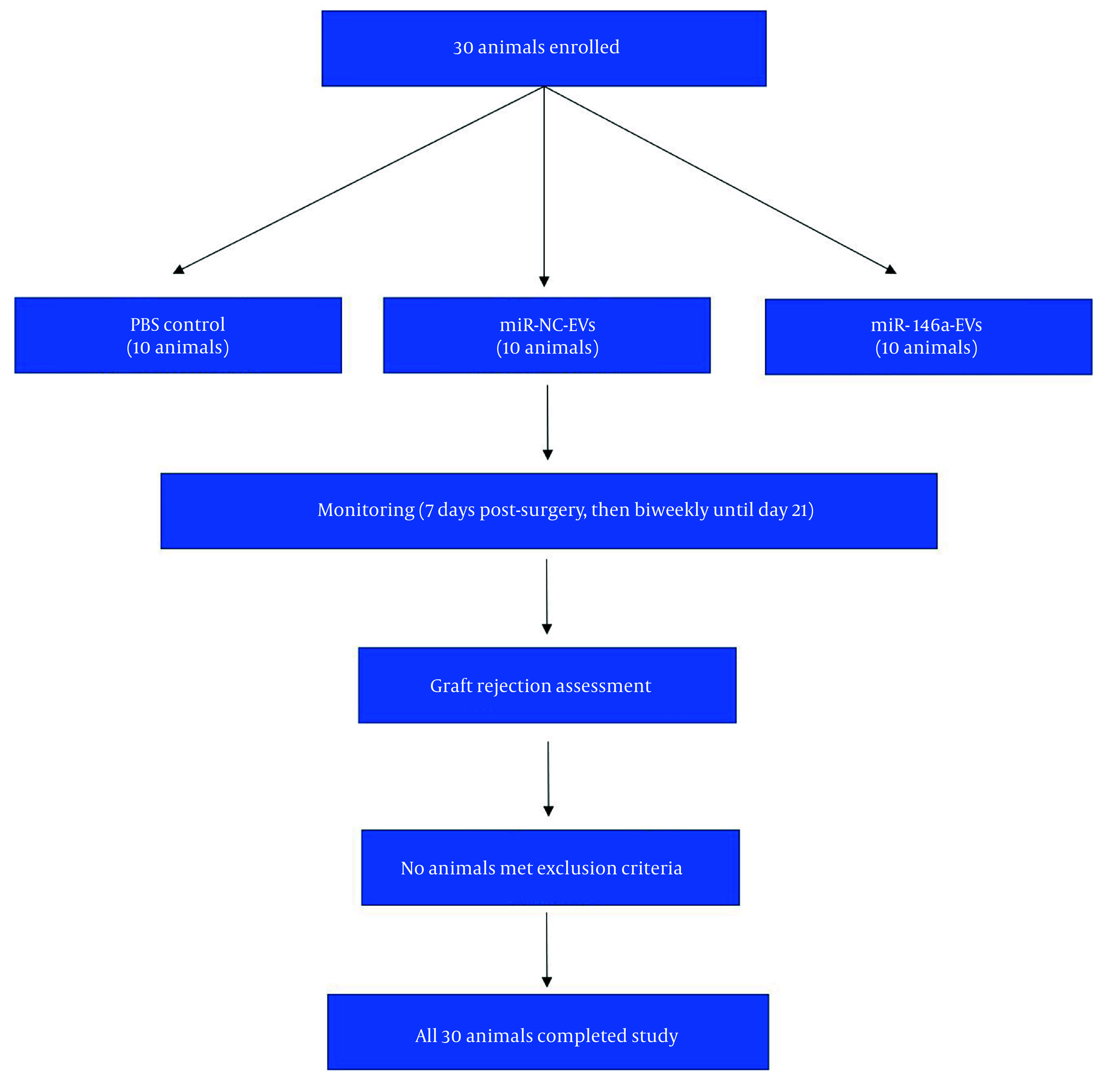
Flowchart of animal progression through the study. Initially, 30 healthy animals were enrolled and randomized into 3 groups of 10 animals each: A PBS control group, a miR-NC-EVs group, and a miR-146a-EVs group. All animals were monitored and assessed throughout the experimental period. No animals were excluded because of complications, infection, or graft integrity loss, and all 30 animals completed the experiment.

Corneal allografting was performed using a well-established murine keratoplasty model as described by Yin et al. (12). In this mouse model, fully mismatched mice were used as recipients and developed a predictable reaction against the grafted cornea. Corneal buttons from C57BL/6 mice were transplanted into BALB/c mice. All mice were male, 8 - 10 weeks old, and weighed 18 - 25 g. Animals were confirmed to be healthy by slit-lamp examination and veterinary inspection before enrollment. All animals were maintained under specific pathogen-free conditions in a controlled room at 24°C with light/dark cycles and access to sterile food and water. After induction of general anesthesia, a central button from the donor cornea was excised and immediately placed in a prepared recipient corneal bed. The graft was fixed in place using microsuturing techniques with nylon, and after surgery, the eye received ophthalmic antibiotic ointment and protective measures. Inclusion criteria consisted of healthy animals with clear corneas. Exclusion criteria included intraoperative complications, postoperative infection, or loss of graft integrity. No animals met the exclusion criteria, and all 30 animals completed the study (Figure 1).

### 3.6. Treatment Protocol

Animals were randomly allocated to 3 treatment groups (PBS control, miR-NC-EVs, and miR-146a-EVs) using a computer-generated block randomization schedule. Each treatment group had a target population of 10 animals to ensure adequate statistical power. Allocation concealment was ensured using sealed, opaque envelopes that were opened only at the time of treatment. The experimental unit was the individual animal for clinical scoring (one transplanted eye per animal) and the individual corneal graft for molecular assays. A single subconjunctival injection (50 µg EV protein in 50 µL sterile PBS; concentration, 1 µg/µL) was administered immediately after transplantation. The same total protein dose and injection volume were used for miR-NC-EVs and miR-146a-EVs. Extracellular vesicle preparations used for dosing were normalized by BCA-quantified protein concentration. Treatment timing was considered optimal for modulating the activity of innate immune cells and antigen-presenting cells around the transplanted tissue and for influencing subsequent activation of alloimmune responses. At 21 days after transplantation, the corneal graft bed and serum were collected for downstream endpoint analyses.

All molecular assays were performed using coded specimens. Personnel conducting RNA extraction, cDNA synthesis, qRT-PCR, and ELISA measurements were blinded to treatment group assignments.

The sample size (n = 10 per group) was determined using preliminary pilot data indicating a mean opacity score difference of 2.0 ± 1.5 between treated and untreated eyes at day 21. Using α = 0.05 and 80% power, the minimum required sample size per group was calculated as 9 animals. Therefore, 10 animals per group were selected to accommodate potential attrition.

### 3.7. Postoperative Monitoring and Clinical Scoring

Transplanted mice were monitored for 7 days after surgery and then biweekly until day 21 using a Topcon SL-7F microscope (Japan) under isoflurane (2 - 3%) sedation. Clinical evaluations were conducted by two independent observers who were blinded to treatment group assignment.

Graft rejection was assessed using a standardized scoring system for 3 parameters: opacity, edema, and neovascularization. Each parameter was graded on a scale of 0 to 4. For opacity (corneal clarity), scores were defined as follows: 0, clear graft with iris details fully visible; 1, mild haze with iris details slightly blurred; 2, moderate haze with iris details visible but obscured; 3, severe haze with the pupil margin barely visible; and 4, completely opaque with no view of intraocular structures. For edema (corneal thickness), scores were defined as follows: 0, no edema and normal corneal thickness; 1, mild stromal thickening; 2, moderate stromal thickening with some stromal folds; 3, severe stromal thickening with prominent folds; and 4, bullous edema or descemetocele formation. For neovascularization (vessel ingrowth), scores were defined as follows: 0, no vessels in the graft; 1, vessels extending to < 25% of the graft radius; 2, vessels extending to 25 - 50% of the graft radius; 3, vessels extending to 50 - 75% of the graft radius; and 4, vessels extending to > 75% of the graft radius. The total rejection score was calculated as a composite score (range, 0 - 12) based on the sum of the opacity, edema, and neovascularization scores. Graft failure was defined as a total score of ≥ 6, consistent with established criteria for moderate to severe rejection in murine corneal transplant models.

Eyes that met graft failure criteria (total score ≥ 6) at any time point were not censored from subsequent longitudinal score assessments. For these eyes, individual parameter scores for opacity, edema, and neovascularization continued to be recorded and contributed to total rejection score calculations at each follow-up visit.

### 3.8. RT-PCR Analysis of Gene Expression in Corneal Graft Bed Tissues

To evaluate the expression of IRAK1, TRAF6, TNF-α, and Foxp3, total RNA was extracted from corneal graft bed samples using a commercial RNA extraction kit according to the manufacturer's instructions, with DNase I treatment to remove genomic DNA contamination. Total RNA was analyzed using spectrophotometric assays to determine RNA concentration and purity. cDNA synthesis was performed using 1 µg of total RNA and a reverse transcription kit. qRT-PCR was performed using SYBR Green Master Mix on an RT-PCR detection system. The amplification program consisted of initial denaturation at 95°C for 10 minutes, followed by 40 cycles at 95°C for 15 seconds and 60°C for 1 minute. Gene expression was quantified using the comparative 2^-ΔΔCt^ method, with results normalized to GAPDH and expressed relative to controls.

### 3.9. ELISA Analysis for Evaluating Cytokine Concentrations

According to the manufacturers' protocols, serum levels of key pro-inflammatory cytokines (IFN-γ and IL-17) and anti-inflammatory cytokines (IL-10 and TGF-β) were quantitatively measured using commercially available high-sensitivity sandwich ELISA kits. At the end of each experiment, peripheral blood was collected from each mouse by cardiac puncture and allowed to clot at room temperature for 30 minutes. Serum was obtained by centrifugation at 2000 × g for 15 minutes at 4°C and stored at -80°C until testing. Serum samples were diluted according to the kit instructions.

### 3.10. Statistical Analysis

Descriptive statistical analyses were performed using IBM SPSS Statistics version 26. Data are expressed as mean ± standard deviation (SD). A P value < 0.05 was considered statistically significant. The Shapiro-Wilk test was conducted to assess normality. For normally distributed data (ELISA data), group comparisons were performed using one-way analysis of variance (ANOVA) with the Tukey post hoc test. For nonnormally distributed data (RT-PCR data), the Kruskal-Wallis test with Dunn post hoc correction was used to compare groups. Graphical representations were created using GraphPad Prism version 8. For opacity, edema, and neovascularization as longitudinal outcomes, 2-way repeated-measures ANOVA was used, with treatment and time as fixed factors and Tukey correction for multiple comparisons. The experimental unit for all clinical outcomes was the animal (n = 10 per group), whereas each harvested graft represented a single biological unit for RT-PCR and ELISA analyses.

## 4. Results

### 4.1. Characterization of hUCSCs

**Figure 2.**
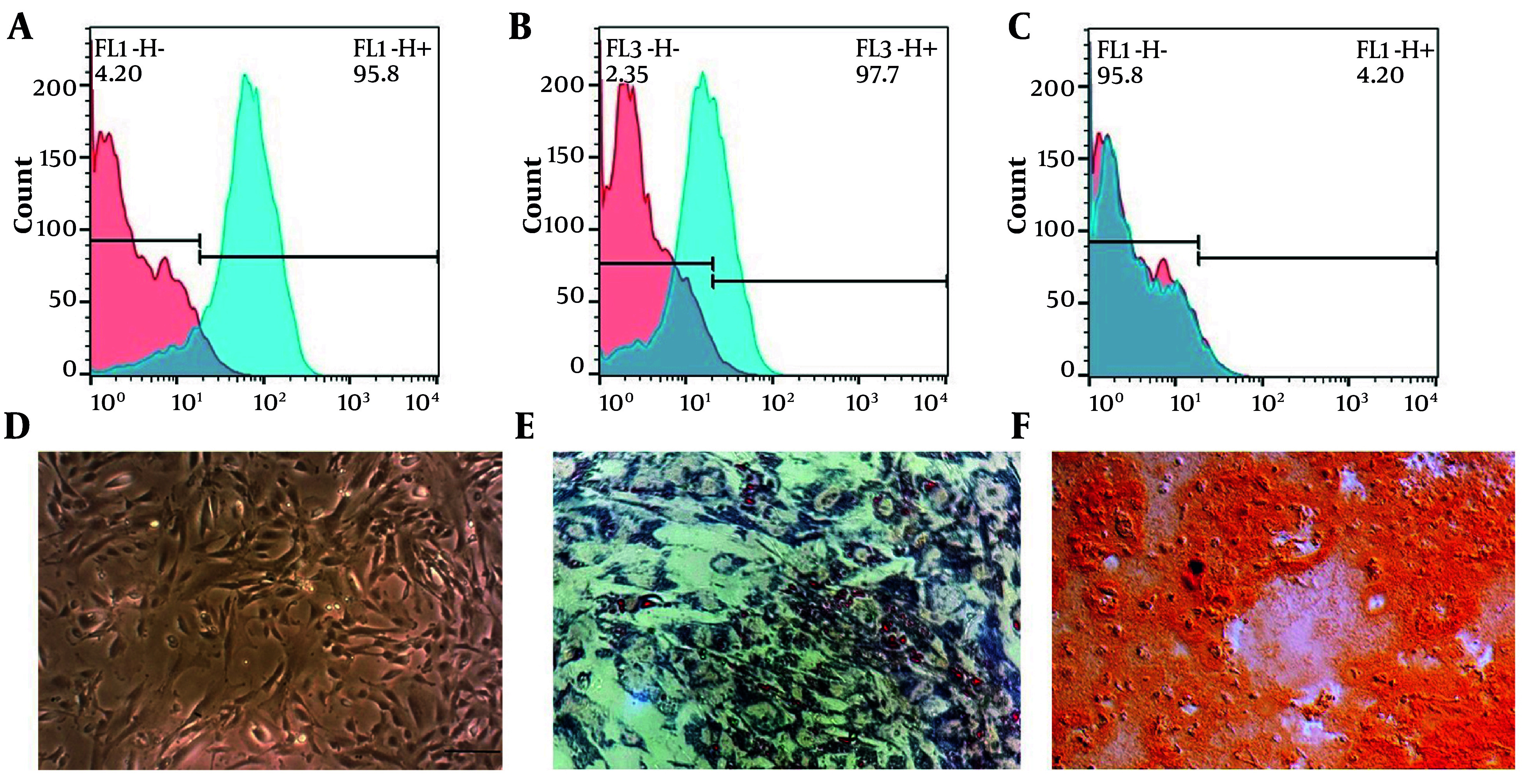
Characterization and multilineage differentiation potential of hUCSCs. A, flow cytometry histogram showing strong positive expression of the mesenchymal surface marker CD90. B, flow cytometry histogram showing strong positive expression of the mesenchymal surface marker CD105. C, flow cytometry histogram confirming the absence of expression of the pan-hematopoietic marker CD45, indicating a lack of hematopoietic cell contamination. D, phase-contrast micrograph of primary hUCSCs in culture, showing characteristic adherent, fibroblast-like, spindle-shaped morphology. E, adipogenic differentiation demonstrated by Oil Red O staining, which highlights intracellular lipid droplets (red/orange) within differentiated adipocytes. F, osteogenic differentiation demonstrated by Alizarin Red S staining, which detects calcium deposits (red/brown) within the mineralized matrix produced by differentiated osteoblasts. hUCSCs: human umbilical cord stem cells.

Flow cytometry demonstrated that these cells fulfilled the minimal criteria for MSC identification. The cells expressed the mesenchymal surface markers CD90 (Figure 2A) and CD105 (Figure 2B). The absence of the hematopoietic surface marker CD45 (Figure 2C) was interpreted as evidence that the cells were not contaminated with hematopoietic cells.

Primary hUCSCs adhered to plastic surfaces and exhibited the expected fibroblast-like, spindle-shaped morphology within 3 - 5 days of culture (Figure 2D). The adipogenic and osteogenic differentiation potential of hUCSCs was demonstrated by their ability to differentiate into adipocytes when exposed to adipogenic-inducing agents and into osteoblasts when exposed to osteogenic-inducing agents. After treatment with adipogenic-inducing agents, the cells developed intracellular lipid-droplet accumulation, as shown by Oil Red O staining (Figure 2E). After exposure to osteogenic-inducing agents, the cells formed highly mineralized matrices that stained strongly with Alizarin Red S (Figure 2F). Collectively, these results demonstrate that hUCSCs exhibited the expected immunophenotype and multilineage differentiation capacity associated with MSCs.

### 4.2. Verification of Successful miR Loading Into hUCSCs

**Figure 3.**
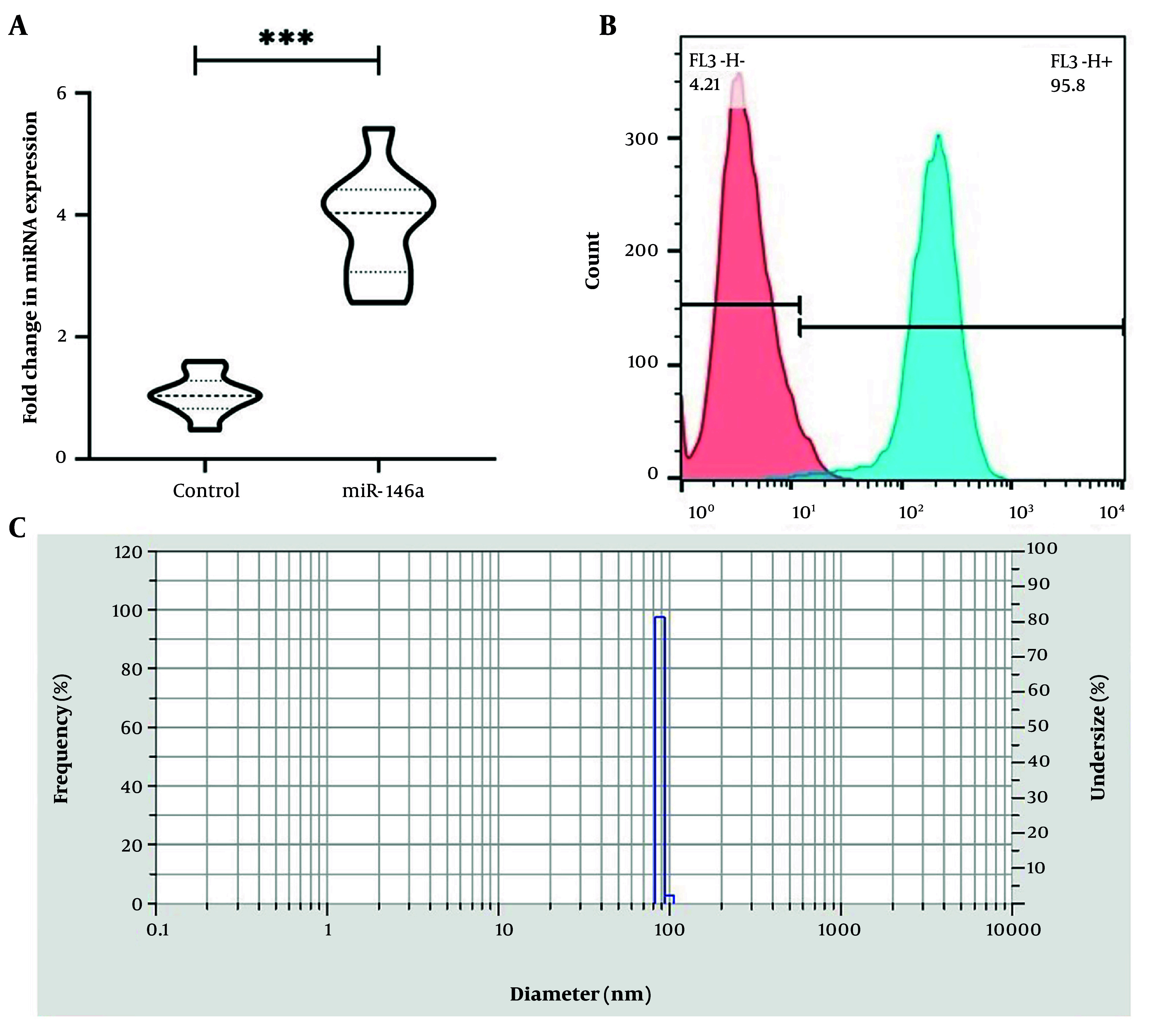
Verification of miR-146a loading into hUCSCs and characterization of derived EVs. A, qRT-PCR analysis confirms significant intracellular upregulation of miR-146a in hUCSCs transfected with miR-146a mimics compared with negative control (miR-NC)-treated cells. B, flow cytometry histogram verifying successful isolation of EVs from hUCSCs, as demonstrated by positive expression of the exosomal surface marker CD9. C, according to DLS analysis using a HORIBA SZ-100 instrument, hUCSC-EVs had an average size of 87.8 ± 1.8 nm. Statistical analysis was performed using the Kruskal-Wallis test with Dunn post hoc test. Data are presented as mean ± SD. *** P < 0.001. Abbreviations: DLS, dynamic light scattering; hUCSCs, human umbilical cord stem cells.

After transfection of hUCSCs with miR-146a mimics, intracellular miR-146a levels increased at 72 hours, indicating successful miRNA delivery using Lipofectamine transfection reagents. qRT-PCR confirmed that miR-146a expression was significantly increased in miR-146a mimic-treated cells compared with miR-NC-treated cells (P < 0.001) (Figure 3A).

### 4.3. Characterization of hUCSC-EVs

After transfection of hUCSCs with miR-146a, EVs were isolated, and CD9 levels were evaluated by flow cytometry. Flow cytometry confirmed the presence of the exosomal marker CD9 in the EV preparation, indicating successful isolation and confirming the exosomal nature of the preparation (Figure 3B). The size and identity of hUCSC-EVs were also confirmed. DLS analysis using a HORIBA SZ-100 instrument revealed an average size of 87.8 ± 1.8 nm (Figure 3C).

### 4.4. Clinical Rejection Scores

**Figure 4.**
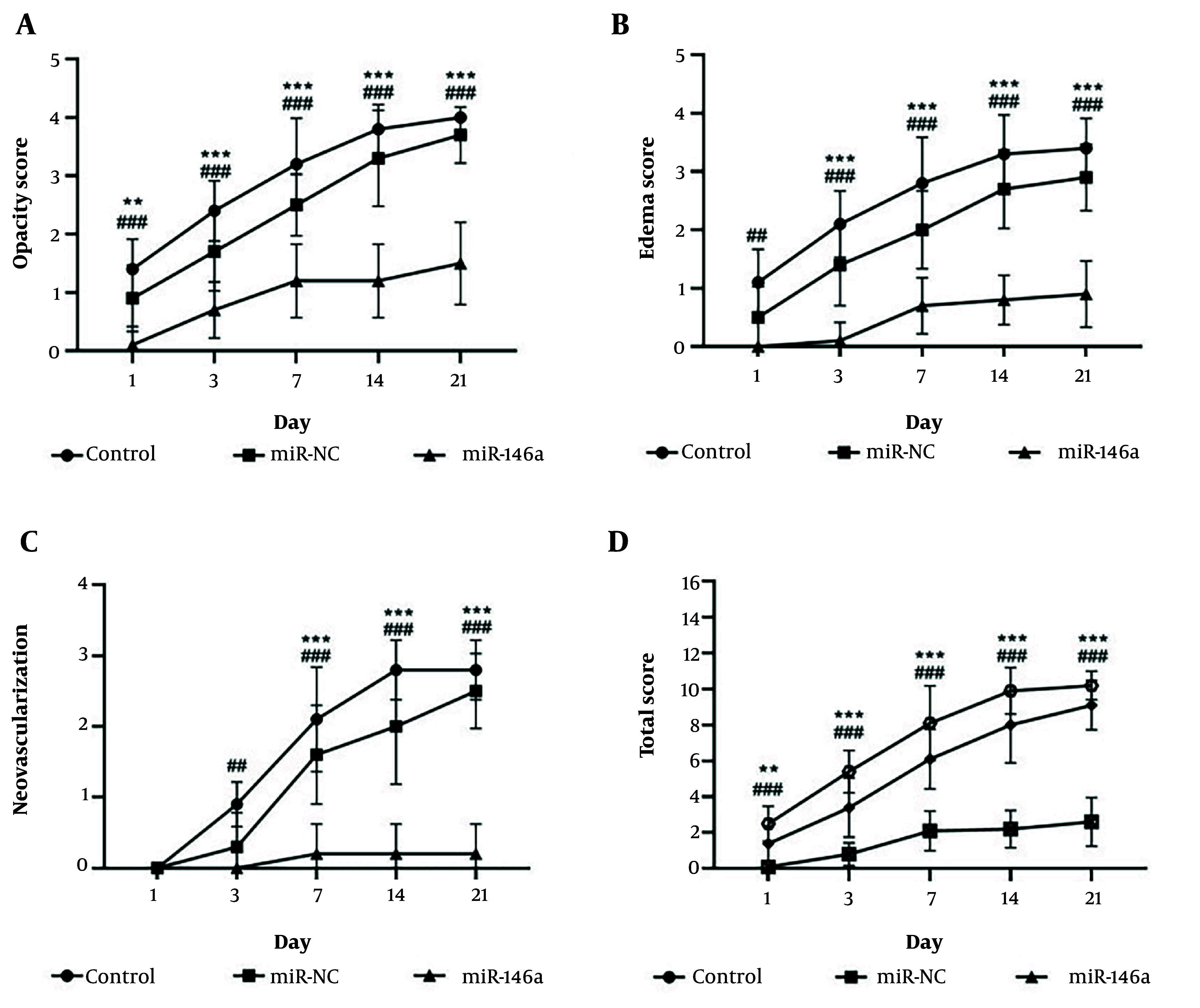
Individual component analysis showing protection across all rejection parameters. A, opacity scores; B, edema scores; C, neovascularization scores; and D, total scores over 21 days. miR-146a-EV treatment significantly reduced all 3 parameters compared with PBS and miR-NC-EV groups. Statistical analysis was performed using 2-way repeated-measures ANOVA (Data represent mean ± SD; n = 10 in each group; ***P < 0.001, **P < 0.01 vs. miR-NC; ###P < 0.001, ##P < 0.01 vs. control).

Total rejection scores (opacity + edema + neovascularization) were significantly reduced in miR-146a-EV-treated mice throughout the observation period (Figure 4). Two-way repeated-measures ANOVA revealed significant effects of treatment, time, and the treatment × time interaction, indicating differential progression of rejection among groups.

At the study endpoint (day 21), total scores were 2.6 ± 1.3 for miR-146a-EV mice versus 10.2 ± 0.9 for PBS mice (P < 0.001) and 9.1 ± 1.3 for miR-NC-EV mice (P < 0.001).

Daily analysis demonstrated that miR-146a-EV treatment provided immediate and sustained protection. Significant differences between miR-146a-EV and PBS controls were apparent by day 1 (P < 0.001) and persisted throughout the study. Compared with miR-NC-EV treatment, miR-146a-EV treatment showed superiority from day 3 onward (P < 0.001). miR-NC-EV treatment provided intermediate protection, with significantly lower scores than PBS on days 3 (P = 0.003), 7 (P = 0.048), and 14 (P = 0.027), but not on day 21 (P = 0.074).

All 3 clinical parameters (opacity, edema, and neovascularization) were significantly improved with miR-146a-EV treatment (Figure 4A-C).

Opacity scores at day 21 were 1.4 ± 0.7 for miR-146a-EV, compared with 4.0 ± 0.0 for PBS control and 3.6 ± 0.5 for miR-NC-EV (P < 0.001). miR-146a-EV-treated corneas maintained near-normal transparency throughout the study, whereas control grafts became progressively opaque.

Edema scores at day 21 were 1.1 ± 0.6 for miR-146a-EV, compared with 3.5 ± 0.5 for PBS control and 2.9 ± 0.6 for miR-NC-EV (P < 0.001). Corneal thickening was minimal in miR-146a-EV-treated mice despite progressive stromal swelling in controls.

Neovascularization scores showed the most pronounced protective effect. miR-146a-EV treatment essentially prevented vessel ingrowth (day 21: 0.1 ± 0.3), whereas the PBS and miR-NC-EV groups developed extensive neovascularization (2.8 ± 0.6 and 2.6 ± 0.7, respectively; P = 0.0002).

### 4.5. Gene Expression Analysis in the Corneal Graft Bed by RT-PCR

To assess the local molecular effects of miR-146a-EV therapy on key inflammatory and regulatory genes, qRT-PCR analyses were performed on RNA extracted from corneal graft bed tissue.

Overall, miR-146a-EV therapy induced marked changes in the graft bed mRNA transcriptome compared with PBS control or miR-NC-EVs. Two genes known to be direct targets of miR-146a and important regulators of the TLR/NF-κB pro-inflammatory signaling pathway, IRAK1 and TRAF6, showed significantly decreased expression after miR-146a-EV treatment compared with the PBS control group (P < 0.001) and the miR-NC-EV control group (P = 0.0165 and P = 0.0410, respectively). In addition, expression of TNF-α, an established pro-inflammatory cytokine, was significantly decreased in the miR-146a-EV group compared with the PBS control group (P < 0.001) and the miR-NC-EV control group (P = 0.0206).

**Figure 5.**
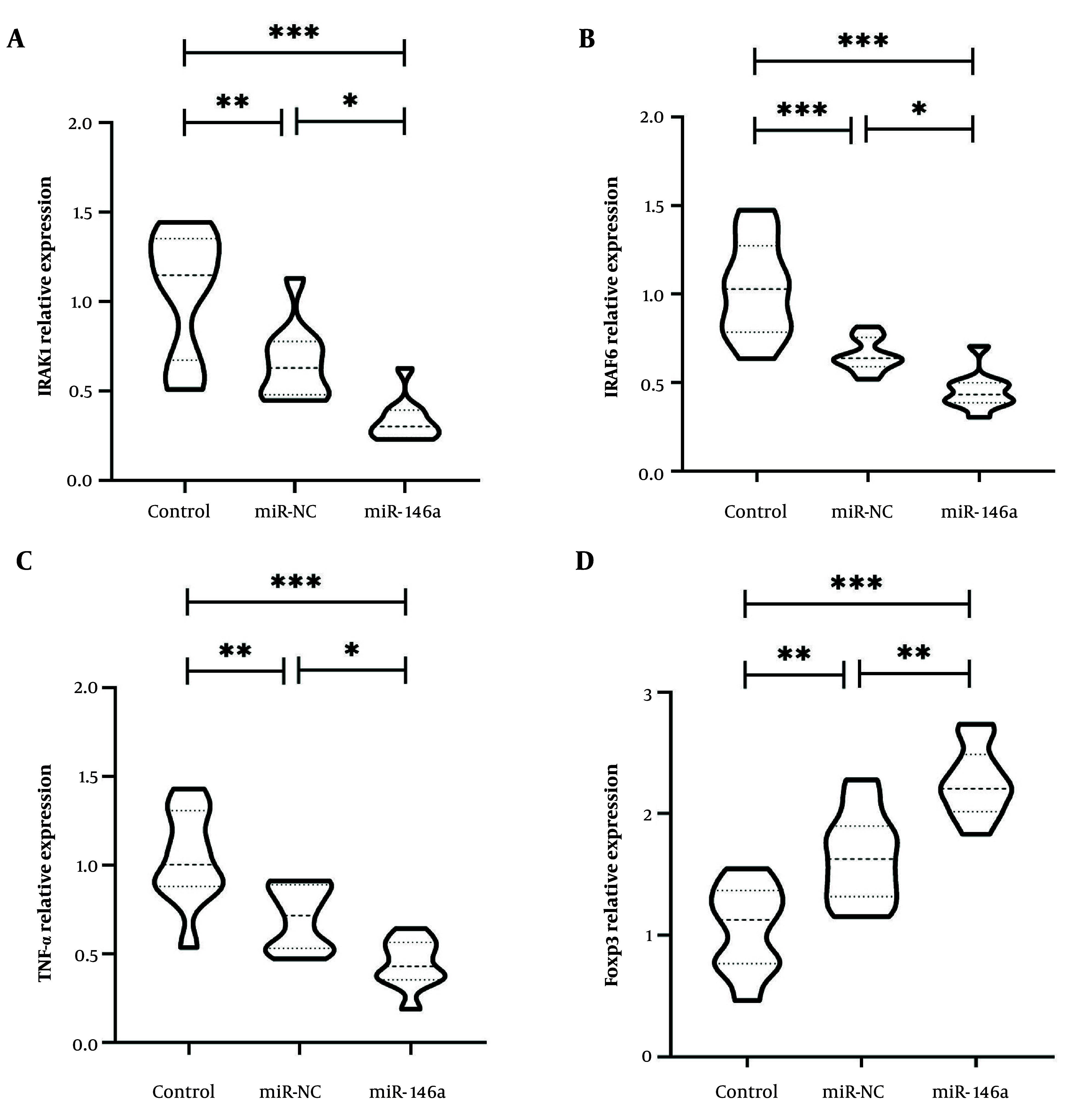
Local molecular effects of miR-146a-EV therapy in corneal graft bed tissue. qRT-PCR analysis shows that miR-146a-EV treatment significantly decreased mRNA expression of the pro-inflammatory genes A, IRAK1; B, TRAF6; and C, TNF-α compared with PBS control and miR-NC-EV controls. Conversely, expression of the regulatory T-cell transcription factor gene D, Foxp3 was significantly increased. Statistical analysis was performed using the Kruskal-Wallis test with Dunn post hoc test. Data are presented as mean ± SD. Statistical significance is indicated as *** P < 0.001, ** P < 0.01, and * P < 0.05. n = 10 in each group. Abbreviations: EV, extracellular vesicle; Foxp3, forkhead box protein 3; IRAK1, interleukin-1 receptor-associated kinase 1; SD, standard deviation; TNF, tumor necrosis factor; TRAF6, TNF receptor-associated factor 6.

By contrast, miR-146a-EV treatment increased expression of Foxp3, the principal transcription factor associated with the development and function of regulatory T cells (Tregs), in corneal tissue from treated mice compared with the PBS control group (P < 0.001) and the miR-NC-EV control group (P = 0.0011) (Figure 5).

### 4.6. Systemic Cytokine Profiling by ELISA

Serum levels of pro-inflammatory and anti-inflammatory cytokines were quantitatively determined by ELISA 3 weeks after transplantation to evaluate the systemic immune response to treatment.

Administration of miR-146a-EVs produced a marked shift in the systemic cytokine profile compared with both the PBS control and miR-NC-EV control groups. ELISA showed that miR-146a-EV treatment significantly decreased serum levels of the pro-inflammatory cytokines IFN-γ and IL-17 and increased serum levels of the anti-inflammatory cytokines IL-10 and TGF-β.

In contrast, mice treated with PBS control or miR-NC-EVs exhibited a strongly pro-inflammatory profile, with increased serum IFN-γ and IL-17 levels and decreased IL-10 and TGF-β levels.

**Figure 6.**
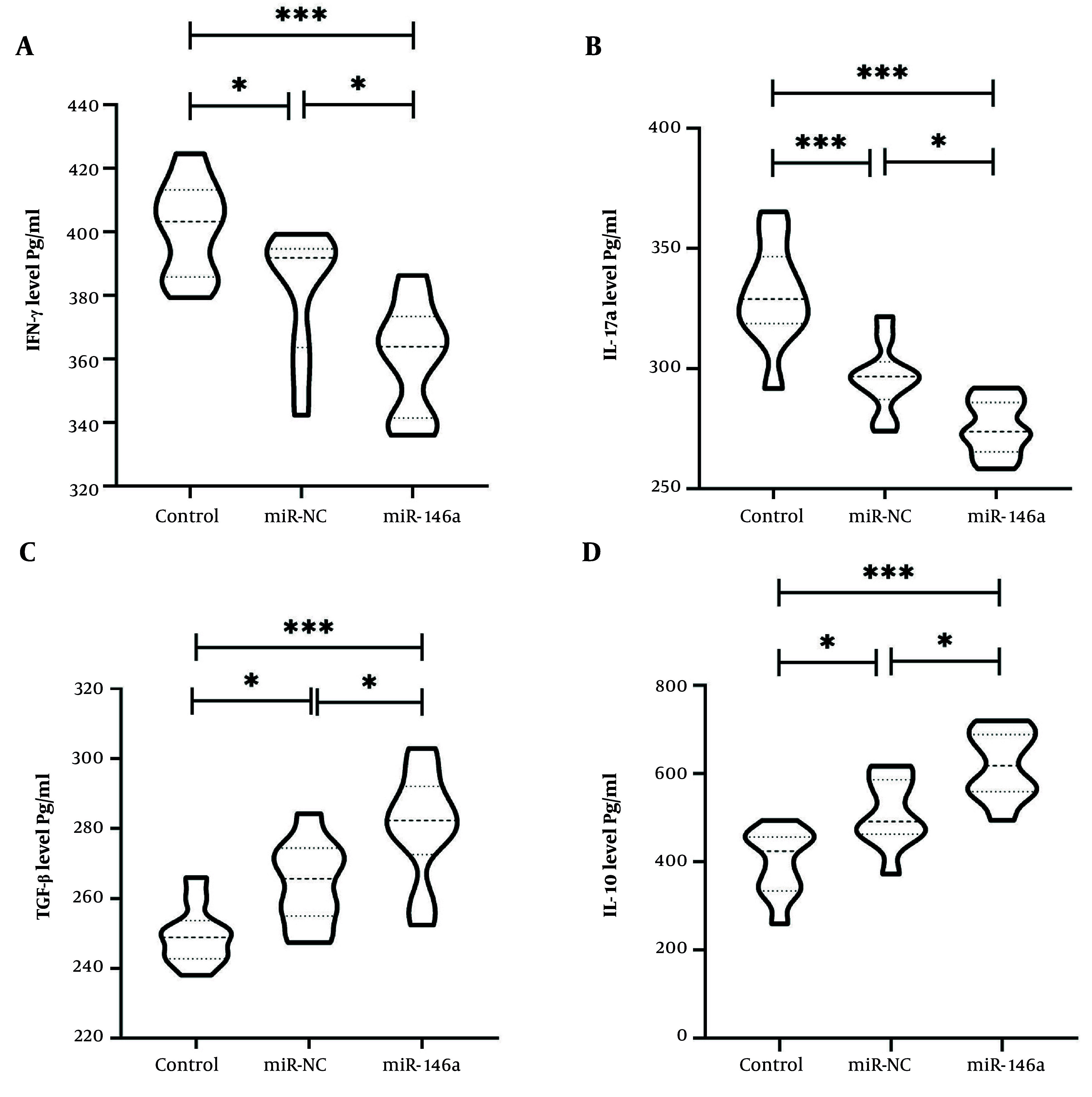
Systemic cytokine profile after miR-146a-EV therapy. Serum ELISA analysis 3 weeks after transplantation shows that miR-146a-EV treatment significantly reduced pro-inflammatory cytokines (IFN-γ [A] and IL-17 [B]) and elevated anti-inflammatory cytokines (TGF-β [C] and IL-10 [D]) compared with PBS control and miR-NC-EV control groups. Statistical analysis was performed using one-way ANOVA with Tukey post hoc test. Data are presented as mean ± SD. Statistical significance is indicated as ***P < 0.001, **P < 0.01, and *P < 0.05. n = 10 in each group. Abbreviations: ELISA, enzyme-linked immunosorbent assay; EV, extracellular vesicle; IFN, interferon; IL, interleukin; SD, standard deviation; TGF, transforming growth factor-beta.

Statistical analysis confirmed significant differences in cytokine concentrations between the miR-146a-EV treatment group and both control groups for IFN-γ, IL-17, IL-10, and TGF-β. Compared with miR-NC-EVs, P values were P = 0.0321, P = 0.0224, P = 0.0108, and P = 0.0172, respectively; compared with PBS control, P < 0.001 (Figure 6). These results demonstrate that local subconjunctival administration of miR-146a-EVs not only alters the local graft environment but also shifts the systemic immune response toward an anti-inflammatory state.

## 5. Discussion

The findings of this study demonstrate that subconjunctival delivery of miR-146a-enriched MSC-EVs exerts both local and systemic immunomodulatory effects that significantly prolong corneal allograft survival. The study also supports the currently accepted model of corneal transplant failure. Previous studies have shown that increased corneal inflammation and dysregulated inflammatory mediators can enhance T-cell stimulation and increase the likelihood of promoting alloreactive immune responses (1). Therefore, a delivery system that reduces early inflammatory signaling may substantially improve corneal transplant outcomes.

The ocular healing potential of MSCs has received increasing attention, with growing evidence supporting the therapeutic effects of both MSCs and their EVs, which are small, biologically active particles released from cells. Kobal et al. reported that MSC-EVs exert anti-inflammatory effects, stimulate epithelial cell healing, and influence local immune microenvironments in the cornea (4). In addition, EV cargo, particularly miRNAs, recapitulates many immunoregulatory activities of MSCs. Dos Santos et al. reported that MSC-EV miRNAs are important signaling mediators that modulate inflammatory signaling pathways and redirect immune responses from pro-inflammatory to anti-inflammatory states (6). Consistent with these findings, the present study showed that miR-146a-EVs inhibit several key inflammatory mediators in the graft bed while inducing a systemic bias toward anti-inflammatory cytokine production.

The substantial decrease in graft opacity and improvement in graft survival observed in the miR-146a-EV-treated group support the established role of immune responses in corneal allograft failure. Corneal rejection is initiated by the activation of antigen-presenting cells, leading to T-cell activation and subsequent production of pro-inflammatory cytokines that cause stromal edema, vascularization, and progressive loss of graft clarity. A meta-analysis of clinical outcomes supports a strong correlation between early inflammation, including opacity and neovascularization, and graft failure, and identifies these inflammatory changes as significant predictors of irreversible corneal allograft rejection (2). Experimental transplantation studies have also shown that activation of innate immunity during the first 2 - 3 weeks after transplantation is a critical determinant of long-term graft survival and that reducing inflammatory responses during this period can enhance long-term graft clarity and survival (12). Collectively, our data confirm that miR-146a-EV treatment significantly reduced early inflammatory damage and produced grafts with longer survival and lower opacity scores, indicating that targeted suppression of inflammatory signaling pathways can alter the postoperative immunologic response and increase graft tolerability.

miR-146a plays an important role in repressing innate immune responses through its effects on the downstream targets IRAK1 and TRAF6, both of which are upstream activators of NF-κB signaling. Zhang et al. demonstrated that miR-146a inhibits the IRAK1/TRAF6/NF-κB pathway, resulting in decreased production of pro-inflammatory cytokines (9). In addition, Abd-Elhalem et al. reported that miR-146a-5p suppresses the TRAF6/IRAK1 pathway and subsequent NF-κB-dependent inflammatory output in a preclinical model of autoimmune disease (10). These prior studies provide mechanistic support for the current study, in which IRAK1, TRAF6, and TNF-α were downregulated in miR-146a-EV-treated grafts. The consistency between these prior findings and the current results strongly supports the conclusion that vesicular delivery of miR-146a can interrupt the initial pro-inflammatory cascade that regulates corneal alloimmunity.

Similarly, a recent study using the systemic administration of MSC-EVs enriched with miR-146a during experimental autoimmune encephalomyelitis (EAE) reported decreased EAE severity, reduced pro-inflammatory cytokines (TNF-α, IFN-γ, and IL-17), increased anti-inflammatory cytokines (IL-10 and TGF-β), and downregulated IRAK1 and TRAF6 expression (7).

Beyond potential therapeutic applications in ocular transplantation, MSC-EVs have been shown to protect photoreceptors from degeneration induced by retinitis pigmentosa by inhibiting microglial activation and inflammatory responses through a miR-146a-dependent mechanism. When miR-146a induces Nr4a3 expression, photoreceptors show significantly increased survival and improved retinal morphology (13).

The present study provides evidence that miR-146a-EVs downregulate inflammatory molecules, including IRAK1, TRAF6, and TNF-α, significantly upregulate regulatory molecules such as Foxp3, and improve graft survival. These results support a mechanistic model in which miR-146a-EVs reprogram the immune response from an effector response toward a regulatory response by modulating both arms of the immune system. This dual modulation of inflammation and tolerance may ultimately prolong corneal allograft survival.

Treatment with miR-146a-EVs in corneal transplantation may enhance Treg induction or recruitment, as suggested by increased Foxp3 expression. Foxp3 is a master transcription factor for T-cell development, and successful transplant outcomes may depend on the presence of Foxp3-expressing Tregs. In many transplantation models, elevated Foxp3 expression has been correlated with improved transplant outcomes (14).

### 5.1. Study Limitations

This study has several limitations. First, although the graft model showed promising outcomes, long-term studies are needed to assess the durability of immune tolerance, the potential for repeated dosing, and possible off-target effects. Second, the optimal MSC source, including bone marrow, adipose tissue, umbilical cord, or corneal stroma, remains unclear. Extracellular vesicle cargo, including miRNAs and proteins, differs according to MSC origin, which may influence immunomodulatory and regenerative efficacy. Third, standardized Good Manufacturing Practice-compliant EV production and purification methods are needed, particularly if clinical translation is pursued, as demonstrated in other fields such as stroke therapy. Finally, although the mechanistic interpretation in this study was based on mRNA expression of IRAK1, TRAF6, TNF-α, and Foxp3, protein-level confirmation and immune cell profiling, such as Treg enumeration and APC phenotyping, were not included. Future studies incorporating Western blotting, flow cytometry, and pathway-level assays will be essential to substantiate the proposed miR-146a-mediated suppression of inflammatory signaling.

### 5.2. Conclusions

Overall, these findings, together with the currently published literature, suggest that MSC-EV therapies enriched with miR-146a represent a promising, biologically rational, noncellular therapeutic approach for preventing corneal graft rejection. Additional preclinical and clinical studies are needed to develop optimal protocols for deriving, harvesting, and evaluating EVs; determining appropriate dosing and timing; and establishing the long-term safety of MSC-EVs for eventual clinical use in corneal transplantation.

## Data Availability

The data that support the findings of this study are available from the corresponding author upon reasonable request.
